# Particle size-dependent quantitative and qualitative differences of common microplastic detection procedures: Nile Red-assisted fluorescence microscopy and confocal micro-Raman spectroscopy

**DOI:** 10.1007/s10661-025-14317-7

**Published:** 2025-07-21

**Authors:** Steve Utecht, Stefan Krause, Tobias Schuetz

**Affiliations:** 1 Spatial and Environmental Sciences, Trier University, Campus II, 54286 Trier, Germany; 2 School of Geography, Earth and Environmental Sciences, University of Birmingham, Edgbaston, Birmingham, B15 2TT UK; 3 Laboratoire d’Ecologie Des Hydrosystemes Naturels Et Anthropises (LEHNA), University Claude Bernard, Lyon1, Lyon, France; 4 BISCA – Birmingham Institute of Sustainability and Climate Action, Birmingham, B15 2TT UK

**Keywords:** Microplastics, Nile Red-assisted fluorescence microscopy, Confocal micro-Raman spectroscopy, Fenton’s reagent, Data validation and correction

## Abstract

Microplastics (MPs) are pervasive and widespread pollutants penetrating ecosystems worldwide, including aquatic environments and sediments. The lack of standardised evaluation procedures and limited sample throughput hampers accurate assessment of global MP pollution. High-throughput analytical methods are crucial for advancing our understanding of MP cycling in the environment. This study compares MP observations by confocal micro-Raman spectroscopy and Nile Red-assisted fluorescence microscopy to evaluate their effectiveness for high-throughput MP analysis using the percentage differences (%DIF) between the results of the two methods. The results show the influence of particle size on the detected percentage differences and demonstrate that both methods deliver better matching results at smaller particle sizes. The overall percentage difference in the number of detected MP counts between the two methods is 421%, with variations ranging over three orders of magnitude depending on morphological characteristics (particles and fibres) and particle size, whereas the analysis of the distribution of the detected polymers across the particle size fractions does not indicate that specific polymer types influence the observed %DIF between the two methods in this study. The combination of the Fenton reagent’s limited organic matter removal and the resulting increased risk of false-positive MP detection, along with Raman spectroscopy’s ability to reliably distinguish MPs from organic components, offers opportunities for data validation and correction to enhance accuracy and reliability of the results. This study contributes to the development of robust methods for high-throughput MP analysis, enabling improved spatial and temporal monitoring of its fate and transport in natural fluxes.

## Introduction

Although pollution by MP (< 5 mm in size) has been evidenced around the globe, there is still a critical lack of understanding the underlying fate and transport mechanisms that control MP occurrence and exposures in the environment (Käppler et al., 2015; Li et al., 2018; Tagg et al., 2015; Thompson et al., 2009). The lack of inter-comparability of results from existing studies contributes to the absence of comprehensive monitoring programs and standardised methods for the assessment of MPs in natural systems (Müller et al., 2020). Analytical techniques frequently used for quantitative and qualitative detections of MP samples such as confocal micro-Raman spectroscopy (cmRs), Fourier-transform infrared spectroscopy (FTIR) or pyrolysis–gas chromatography-mass spectrometry (pyro-GC–MS) show high detection accuracy but come at high instrumental and staffing cost (Shim et al., 2016; Sturm et al., 2021; Vašková, 2011).

The cmRs is usually considered as a robust technique for the detection of MP offering high precision quantitative and qualitative measurements of a wide range of polymer types (Anger et al., 2018; Chakraborty et al., 2023; Dąbrowska, 2021; Sobhani et al., 2019; Xu et al., 2019). In addition, there is high certainty in separating organic matter detections from the data, as the obtained spectra do not coincide with plastic spectra.

The exclusive use of cmRs for MP detection results in considerably extended measurement times. This limitation hampers the feasibility of high-throughput MP analyses necessary for handling larger sample quantities. Other limitations include susceptibility to spectral fluorescence interference, the analytical detection limit of 1 μm, weak or altered signals from solid samples due to natural-related modifications and the lack of free available reference libraries (Ivleva, 2021; Shim et al., 2017; Wirnkor et al., 2019), Hence, the scope of studies aiming at MP detection is often limited by the restricted number of samples that can be analysed in a reasonable timeframe (Xu et al., 2019). Addressing this critical limiting factor, more efficient methodological approaches that take into account simplicity in application, time and cost efficiency, as well as accuracy, reliability and repeatability for the detection of MP from environmental samples are urgently needed (Abusafia et al., 2023; Hengstmann & Fischer, 2019; Hurley et al., 2018; Jain et al., 2018; Prata et al., 2019a; Renner et al., 2019; Tagg et al., 2017).

Because of its ease of use, the fluorescent dye Nile Red (NR) in combination with fluorescence microscopy is frequently used as an inexpensive, time-efficient and widely accessible method for the quantification of MPs (Maes et al., 2017; Meyers et al., 2022; Nel et al., 2021; Patchaiyappan et al., 2020; Sancataldo et al., 2020; Shim et al., 2016). Many pristine plastic polymer types stain reasonably well with NR across different particle sizes, ranging from a few nanometres up to several centimetres, generating high-intensity NR signals that can be efficiently detected by fluorescence microscopy (Bianco et al., 2023; Carter et al., 2023; Cole, 2016; Kang et al., 2020; Tamminga, 2017; Zhang et al., 2023). Besides that, NR-dyed samples spiked with known quantities of MP show high recovery rates, proving that Nile Red-assisted fluorescence microscopy (NRafm) is an efficient MP detection method (Pan et al., 2023; Shim et al., 2016; Stanton et al., 2019). Additionally, the Nile Red staining method has been successfully combined with spectroscopic techniques such as Raman spectroscopy, FTIR spectroscopy, flow cytometry, photoluminescence excitation (PLE) spectroscopy, and optical photothermal infrared (O-PTIR) spectroscopy to verify fluorescently labelled objects as plastic particles (Barrett et al., 2020; Bianco et al., 2023; Erni-Cassola et al., 2017; Konde et al., 2023; Maes et al., 2017; Shim et al., 2016; Sturm et al., 2021; Vermeiren et al., 2020). The robustness of the use of the Nile Red staining method for detection of MP without verification of at least a subsample of identified particles by any of the aforementioned spectroscopic methods has been questioned (Shim et al., 2016).

The combination of spectroscopic MP detection techniques and the Nile Red staining method enables the analysis of larger sample volumes, with Nile Red staining facilitating high-throughput screening through rapid application, while spectroscopic approaches can be applied to a subset of the samples to ensure accurate polymer identification. In this context, De Guzman et al. (2022) report an average percentage difference of 117.03% between the Nile Red staining method and their micro-FTIR analysis, while Stanton et al. (2019) investigated the potential overestimation of MP particles by comparing Nile Red staining results with another dye (4′,6-diamidino-2-phenylindole), which binds to biological materials, finding that Nile Red staining method alone led to a 100% overestimation of MP particles.

Recent developments in the interpretation of photographic Nile Red fluorescence images (pixel brightness) for separating organic matter from MP, along with the polymer-specific fluorescence behaviour enabling discrimination between different plastic types, represent promising approaches for MP detection. These advances may allow for rapid and robust identification of polymer types and effective separation of MP from organic material, both of which are essential for obtaining reliable and reproducible results (Kukkola et al., 2023; Sancataldo et al., 2020). However, photographic (NR-) fluorescence images show that organic matter and MP can emit similar light intensities indicating that the presence of organic matter in environmental samples may limit the detection accuracy of MPs (Konde et al., 2023; Sturm et al., 2021, 2023). In addition, the use of organic solvents in the NR staining procedures of MPs carries the risk of overlooking organic solvent-induced morphological modifications, as shown by Tamminga, (2017) and Kang et al. (2020) for polystyrene for instance. In an unpublished experiment, we determined a weight loss of 1% after 5 min and 7% after 20 min for PMMA (*d* = 2 mm) when exposed to an acetone-ethanol mixture (50:50). This limits the use of NR as a standalone tool for the detection of MP from natural systems. Both, the inadvertently labelling of residues of organic matter and potentially organic solvent-induced modifications of MPs, contribute to the generation of false positives MP detections. Considering the wide range of MP particle sizes in environmental samples, the extent to which these limitations affect different MP size classes and morphologies is not well understood To perform reliable high-throughput MP analyses of environmental samples, it is essential to evaluate the streamlining of a dual-method MP detection approach combining cmRs and NRafm, which may enable the transition from local point sampling and smaller regional studies to larger-scaled measurement campaigns (Liu et al., 2022; Range et al., 2023; Shim et al., 2016; Shruti et al., 2022).

Therefore, this study aims (1) to systematically evaluate detection discrepancies between cmRs and NRafm by analysing the percentage differences (%DIFs) in the number of detected MP counts from 100 environmental samples across five selected particle sizes (5000–25 µm). Furthermore, it aims (2) to assess morphology-dependent detection accuracy by comparing the reproducibility of morphological features (particles or fibres) identified through both NRafm and cmRs methods. The overall ambition of this study is to contribute to the ongoing development of suitable MP detection methods for high-throughput field campaigns that can more reliably capture the nature of MP environmental fate and transport through the analysis of representative sample quantities.

## Materials and methods

### Extraction of MP from river sediment samples

To account for the representativeness and complexity of environmental samples in this study and to better identify potential sources of analytical error, natural sediment samples were used for the analyses. Microplastics in environmental samples can differ significantly from artificially produced laboratory samples, as they are subject to environmental aging (Waldschläger et al., 2022). Replicating the exact impacts of environmental aging mechanisms on MP is challenging when using artificially altered laboratory samples carrying the risk of overlooking naturally occurring random effects or complex particle–matrix interactions. The MP samples used in this study were collected as part of an investigation studying the transport and deposition of MP in the riverbed sediments. A total of twenty frozen sediment cube samples (SCS with a volume of 125 cm^3^ and an edge length of 5 × 5 × 5 cm. The total volume consisting of 20 SCSs corresponds to 2500 cm^3^) were cut from two frozen sediment cores obtained at the Ruwer, a gravel bed tributary of the river Moselle near Trier in southwest Germany. The cores were extracted using the freeze-core sampling method with liquid nitrogen as a coolant (Hauer et al., 2020; Straßer et al., 2014). The catchment area is located within the Rhenish Massif, which is primarily characterised by shale rock. As described below, each sample was fractionated into sub-samples encompassing five particle sizes ranging from 5000 to 25 µm, which resulted in a total of 100 samples.

**Fig. 1:**
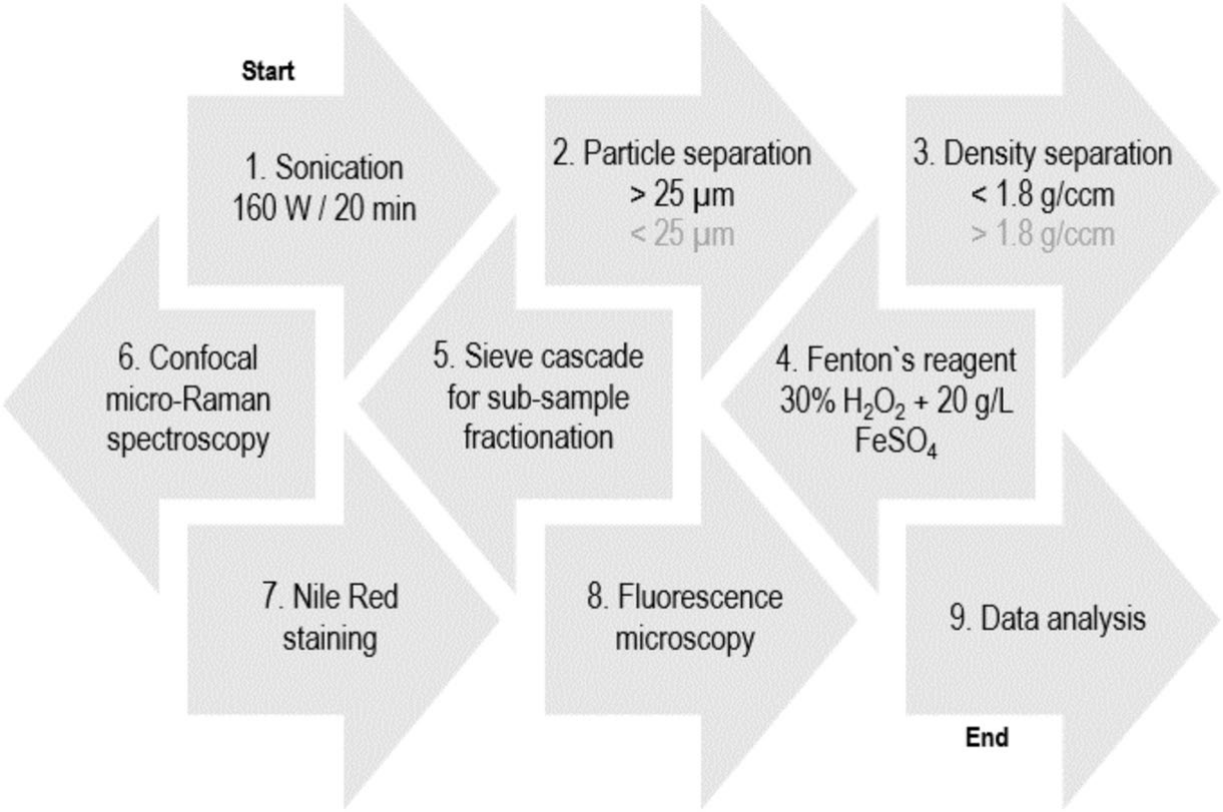
Flow chart of pre-treatment flow strategy (1–9) for the extraction of MP from environmental samples adopted in this study

First, all sediment samples were placed in glass vessels and submerged in deionized water. The samples were sonicated to break up sedimentary agglomerations (Fig. 1). Von der Esch et al. (2020) sonicated polylactic acid (PLA), polystyrene (PS), and polyethylene terephthalate (PET) for 15 h at 35 kHz in MilliQ water, observing changes in morphology at the nanometre scale (e.g. surface and edge modifications) as well as in chemical composition (e.g. OH, C = O and COOH groups). Therefore, the samples were exposed to sonication for 20 min at 35 kHz and 160 W, solely to break up sedimentary agglomerates ensuring that all components of the sample matrix were present as individual particles (Büks et al., 2020). Particles with a diameter smaller than 25 μm were isolated by sieving from the samples. Next, density separation was performed using sodium polytungstate (SPT-3) (TC-Tungsten Compounds) with a density of 1.8 g/cm^3^. During density separation, attention was paid to the economic use of the separation fluid. Due to differences in sedimentation rates based on grain size, fractions smaller than 250 μm were treated separately from those larger than 250 μm. Preliminary tests revealed that particles (> 250 μm) sedimented immediately, while smaller particles (< 250 μm) took significantly longer. Therefore, pre-sorting of the particles proved to be advantageous, as (a) the sample material was reduced, facilitating better handling and (b) the particle size fraction larger 250 μm could be density-separated without additional resting time. For the > 250 µm fractions, density separation was carried by placing them in a crystallization dish (Ø 90 mm; 300 ml), covering the sample with SPT-3 and stirring with a spatula. Floating material was immediately decanted and collected in a 250 µm sieve. The separation of the particle size fractions (< and > 250 µm) further simplified handling during density separation, as the separation fluid (SPT-3) remained largely clean when separating particles larger than 250 μm, facilitating its reuse. The decanting process was carried out according to Imhof et al. (2012). This process was repeated at least three times until no visible floating material remained, ensuring high recovery rates. In the < 250 µm fractions, density separation was performed by placing them in a smaller crystallization dish (Ø 70 mm; 100 ml). After covering the samples with SPT-3 and stirring, the samples were left to rest in a refrigerator for 24 h. After resting, the samples were decanted and collected in a 25 µm mesh sieve. Due to the long sedimentation time, this procedure was performed once, which may have contributed to a reduced recovery rate, potentially further influenced by the small particle size (Enders et al., 2020; UguagLiati et al., 2024).

Following density separation, both < 250 and > 250 μm fractions were cleaned with deionized water and oven-dried at 50 °C for 4 h. Subsequently, the sample fractions were recombined to remove organic matter through digestion using Fenton’s reagent (30% H_2_O_2_ + 20 g/l FeSO_4_, pH = 3) in a 500 ml beaker. First, FeSO_4_ was added to a 500-ml beaker, followed by the sample. Then, H₂O₂ was added in five portions of 10 ml each. Based on internal laboratory experience with the Fenton reaction, a time efficient modification was introduced by accelerating the addition of H₂O₂, using five portions of 10 ml each instead of the originally proposed ten portions of 5 ml (Al-Azzawi et al., 2020). This adjustment requires the use of a 500-ml beaker.

There are conflicting results regarding the effects of prolonged exposure to the Fenton reaction solution on different polymer types. However, recent studies suggest that prolonged exposure at elevated temperatures can increase solubility and promote the degradation of certain polymers, such as PA and PET (Al-Azzawi et al., 2020; Li et al., 2023; Vojnović et al., 2024). Therefore, in this study, the exposure time in the Fenton reaction solution was limited to 15 min to minimise the potential degradation of chemically unstable polymers (Prata et al., 2024). Upon completion of the reaction procedure, the sample was transferred to a 25 µm sieve and cleaned with deionised water to remove residual chemicals (Al-Azzawi et al., 2020; Campo et al., 2019; Frei et al., 2019; Hu et al., 2022; Hurley et al., 2018; Kabir et al., 2022). Finally, the samples were fractionated into five selected particle sizes (630–5000 µm (> 630 µm), 250–630 µm (> 250 µm), 125–250 µm (> 125 µm), 75–125 µm (> 75 µm) and 25–75 µm (> 25 µm)) using sieve cascades. The sample weights were measured to estimate the number of particles on each filter membranes.

### Semi-automatic confocal micro-Raman spectroscopy

For the cmRs-based qualification and quantification, the particle size fractions > 250 µm were placed centrally on a glass plate equipped with a graph paper (Fig. 7C) and evenly distributed by gently tapping from the underside before being spectroscopically measured. Composite microscopic imaging stacks of the samples were recorded to obtain the coordinates of the objects on the glass plate, enabling semi-automatic measurements using the Sample Raster feature in WITec Control 4.1 software.

The particle size fractions < 250 µm were homogenously transferred onto glass fibre (GF) filter membranes (Macherey–Nagel GF-2, Ø 45 mm, pore size 0.5 µm) using vacuum filtration and subsequently dried in an oven at 50 °C. A portion of the filter membranes comprising 49.76% (2.5 cm × 2.5 cm with an area of 6.25 cm^2^) of the total filter membrane was microscopically mapped to locate the objects (Objective: Zeiss EC Epiplan-NEOFLUAR 10 × magnification) before qualitatively and quantitatively measured using cmRs (WITec alpha 300 R; Operating system: WITec Control 4.1).

Within the mapped area of the filter membranes, a maximum of 400–500 particles were selected, a process that took approximately 1 h. It should be noted that the portion of the area required for selecting 400–500 particles decrease relatively to the total mapped area of the filter membrane as particle size decreases. Once the maximum number of particles was reached, the portion of the area occupied by the selected particles was estimated as a percentage and extrapolated to the total filter membranes (12.56 cm^2^) (e.g., Käppler et al., 2016; McCormick et al., 2016; Xu et al., 2019).

A total of 7.97 g (0.18%) of the total dry weight of the sediment cubes (4.52 kg) was extracted by density separation and used for the subsequent pretreatment process. Depending on the particle size fraction, 7.70 g (97%) was analysed on glass plates (> 250 µm) and glass fibre filter membranes (< 250 µm) using cmRs, corresponding to 34,538 spectroscopically measured objects. To assess the representativeness of the Raman measurements, the total dry mass of the density-separated samples was used as a reference and compared with the actual sample mass analysed under the Raman microscope. It was found that the spectroscopically measured mass fractions of the density-separated samples can differ by up to two orders of magnitude from their total mass, thereby reducing representativeness, particularly for smaller particle sizes (< 250 µm).

For particle detection, a 532 nm laser with laser intensity of 4 mW was used. A single spectral scan was performed with an integration time of 10 s and one accumulation, resulting in measurement durations of approximately 3 h. All recorded spectra were visually inspected and potential MP spectra were manually re-measured with variable laser intensities (0.1 to 15 mW) to improve quality for comparison with a reference library.

### Nile Red application and acquisition

After applying the cmRs, particle larger than 250 µm were placed in a drilled notch of a piece of aluminium holder and covered with NR solution at a concentration of 1 mg/ml (Tamminga, 2017) in a chloroform-acetone mixture (3:1). The NR solution was allowed to fully evaporate. Subsequently, the fluorescence-labelled particles (> 250 µm) were evenly distributed on a glass plate and illuminated with blue LED light (~ 470–475 nm). All NR-labelled particles were manually counted using a magnifying glass and a blue bandpass filter (orange foil) to evaluate differences in the number of MP counts between cmRs and NR staining (Konde et al., 2020; Prata et al., 2019b).

The smaller particle sizes (< 250 μm) were stained with NR directly on the filter membranes by applying the dye dropwise. Three mapping areas, measuring 0.83 cm × 0.83 cm and covering an area of 2.07 cm^2^, were analysed. After recording using the confocal Raman microscope, the results were extrapolated to represent the total filter membrane. To optimise sample illumination, three blue light LED flashlights were positioned around the microscope. During fluorescence imaging, the objective was equipped with a blue bandpass filter attached to the 10 × magnification objective. The room was darkened, and the monitor was switched off to minimise interference from external light sources. To improve visibility of darker areas of the fluorescence images, the contrast was enhanced by at least 75% for all images. All NR-stained observations were manually counted. The counting procedure was performed twice and the mean number was used for further analysis. Further details on the potential of organic solvents used during pretreatment to degrade certain polymer types and thus influencing the detectability of the fluorescence response are addressed later in the discussion.

The detected MP counts, microplastic particles (MPP) and microplastic fibres (MPF) of both methods were analysed for significant differences. Based on the average length and width of a sample of 20 fibres, a minimum length-to-width ratio of 30:1 was defined for fibre classification. The variation in the detected MP counts was quantified using the percentage difference (%DIF), calculated according to the formula provided below.

1 $$\% \mathrm{DIF}=\left(\frac{\text{Higher MP counts}-\text{Lower MP counts}}{\text{Lower MP counts}}\right)\times 100$$

### Data analysis

MS Office programs were used to visualise the data. The non-parametric Wilcoxon signed-rank test was applied at a significant level of 0.05 (95% confidence interval) to determine whether the total detected MP counts, MPP and MPF, including all selected particle sizes, differed significantly between the cmRs and NRafm methods.

## Results

**Fig. 2:**
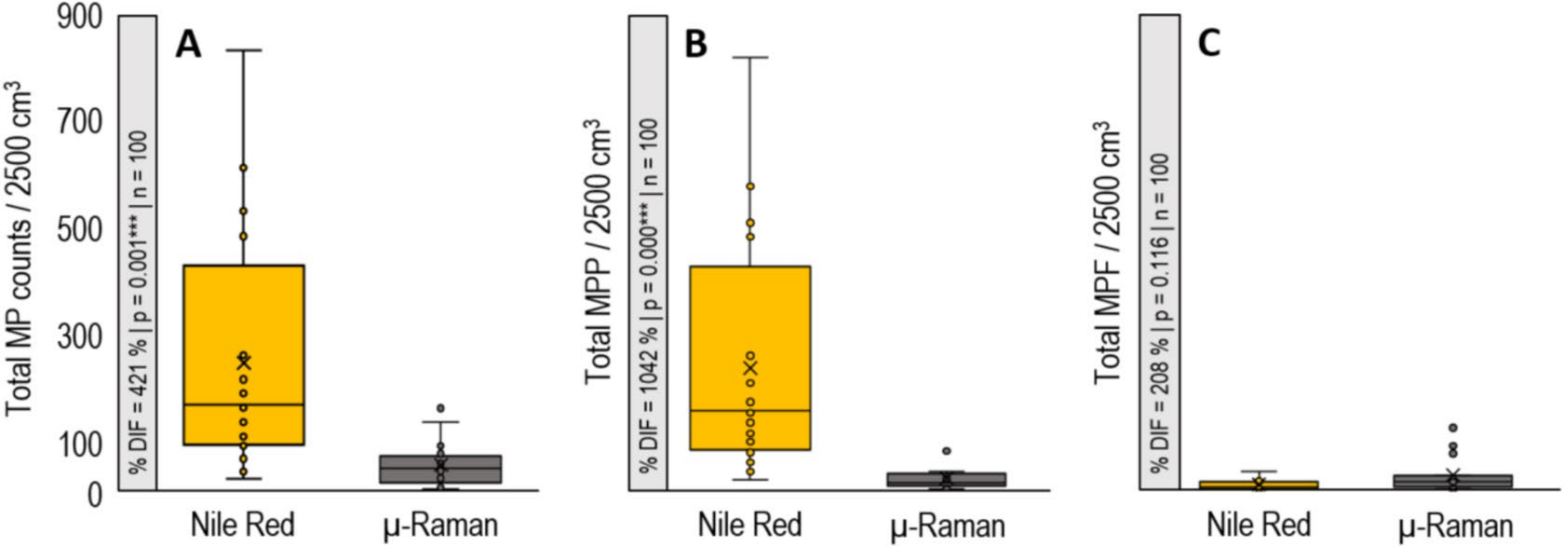
**A** Nile Red-assisted fluorescence microscopy (yellow) and confocal micro-Raman spectroscopy (grey) total microplastic (MP) counts from 20 sediment cube samples (*Volume*_Total_ = 2500 cm^3^). **B** Total microplastic particles (MPP) and **C** total microplastic fibres (MPF) including percentage differences (%DIF) between both methods, *p*-value (*p*) derived from Wilcoxon signed-rank test (*p* = 0.05) using the total MP counts per sediment cube sample and the number of samples (*n*). Each point in the box plot represents MP counts compiled from five particle size fractions

Out of the 100 samples analysed, the comparison between cmRs and NRafm reveals similar detection results in 8% and dissimilar detection results in 92% of the cases. Using NRafm, the total detected MP counts are 4792 ± 69 MP, with total MPP at 4625 ± 68 and total MPF at 167 ± 3. In contrast, cmRs shows a different detection performance, capturing 920 ± 19 total MP counts, with 405 ± 10 for total MPP and 515 ± 15 for total MPF. This leads to percentage differences (%DIFs; Eq. (1)) between both methods ranging over two orders of magnitude for total MP counts (421%; Fig. 2A), total MPP (1042%; Fig. 2B) and total MPF (208%; Fig. 2C). The distribution of detected MP based on morphological characteristics shows that NRafm yields higher observations for total MP counts and total MPP compared to cmRs, while cmRs reports higher numbers for total MPF. The non-parametric Wilcoxon signed-rank test (*p* = 0.05) confirmed significant differences between the methods for total MP counts and total MPP (Fig. 2B, C). However, no significant difference was found for total MPF (Fig. 2A).

The result for MP counts across the particle size fractions reveal that the particle distributions observed with the two methods tend to show similar trends. Both methods demonstrate consistent detection patterns, with fewer MP detections observed for larger particle sizes and higher MP detections for smaller particle sizes. However, this does not apply to the detected MPF, as the number of NRafm-based detections stagnates for particle sizes < 250 µm.

**Fig. 3:**
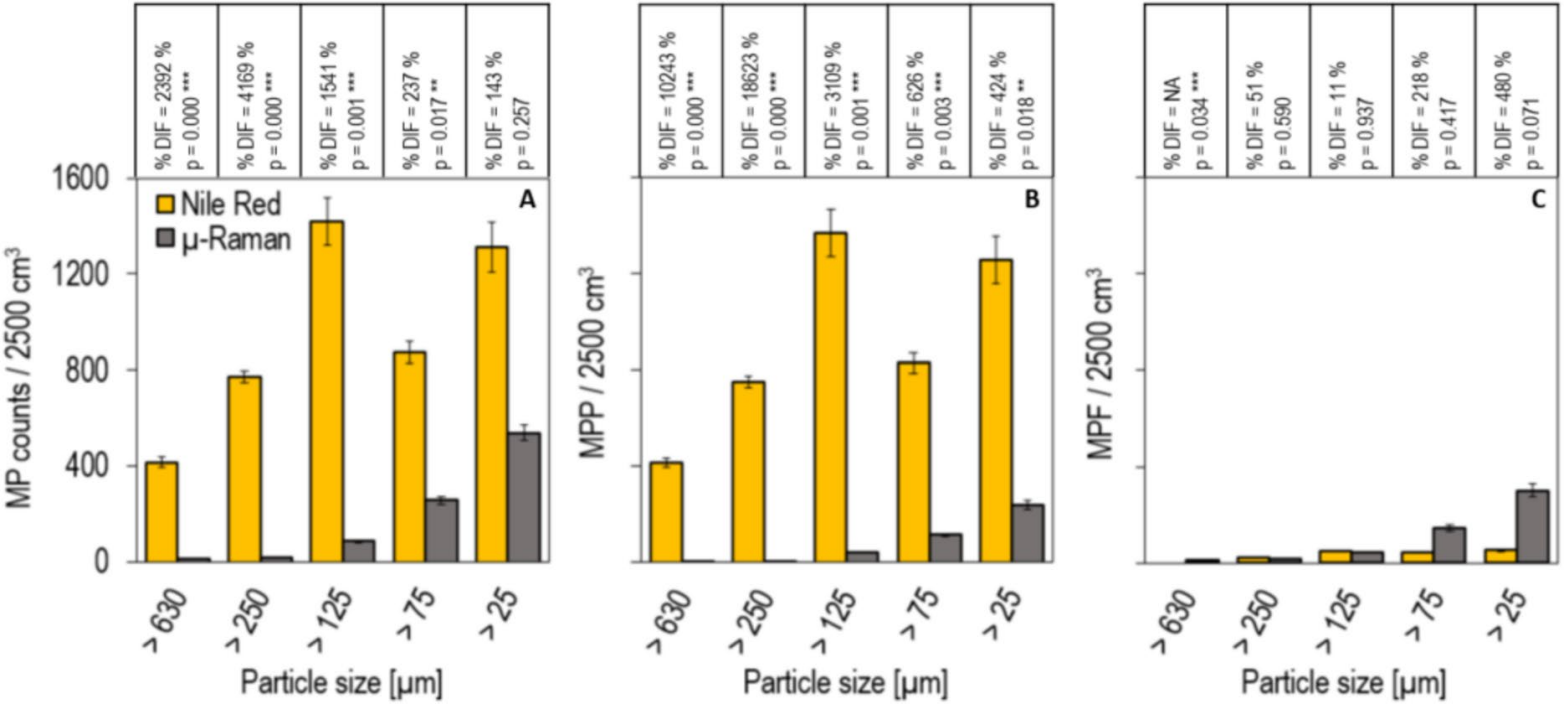
**A** Nile Red-assisted fluorescence microscopy (yellow) and confocal micro-Raman spectroscopy (grey) observed microplastic (MP) counts from 20 sediment cube samples (*Volume*_Total_ = 2500 cm^3^) across five selected particle sizes. **B** Microplastic particles (MPP) and **C** microplastic fibres (MPF) including tabular representation of the percentage differences (%DIFs) for MP, MPP and MPF counts between both methods and the *p*-value (*p*) derived from Wilcoxon signed-rank test (*p* = 0.05) using MP detections per sediment cube sample and particle size. Each bar graph represents 20 observations

The detected MP counts per particle size (Fig. 3A) observed through NRafm are 416 ± 20 (> 630 µm), 774 ± 24 (> 250 µm), 1417 ± 98 (> 125 µm), 874 ± 46 (> 75 µm) and 1311 ± 102 (> 25 µm). In comparison, the detected MP counts observed through cmRs are 17 ± 2 (> 630 µm), 18 ± 2 (> 250 µm), 86 ± 3 (> 125 µm), 259 ± 16 (> 75 µm) and 540 ± 34 (> 25 µm). Therefore, MP counts detected through NRafm exceed those observed by cmRs for all particle sizes. The %DIFs in MP counts per particle size between the two methods show a decreasing trend the smaller the particle size, ranging from 143% (> 25 µm) up to 4169% (> 250 µm). The non-parametric Wilcoxon signed-rank test (*p* = 0.05) indicates significant differences in MP counts between both methods for all particle sizes, except for particles smaller 25 µm.

The morphological evaluation of NRafm-based observations (Fig. 3B) shows consistently higher detections across all particle sizes for MPP: 416 ± 20 (> 630 μm), 753 ± 24 (> 250 μm), 1369 ± 97 (> 125 μm), 829 ± 45 (> 75 μm) and 1260 ± 99 (> 25 μm). In comparison, cmRs-based detections are continuously lower with 4 ± 1 (> 630 μm), 4 ± 1 (> 250 μm), 43 ± 3 (> 125 μm), 114 ± 8 (> 75 μm) and 240 ± 20 (> 25 μm). The %DIF in the number of detected MPP per particle size between the two methods tends to decrease the smaller the particle size ranging from 424% (> 25 µm) up to 18,623% (> 250 µm). The non-parametric Wilcoxon signed-rank test (*p* = 0.05) indicates significant differences between both methods for MPP for all particle sizes, except for particles larger 25 µm.

NFafm-based MPF detections are 0 ± 0 (> 630 µm), 21 ± 2 (> 250 µm), 49 ± 4 (> 125 µm), 46 ± 3 (> 75 µm) and 52 ± 5 (> 25 µm) in comparison to cmRs-based detection with 13 ± 1 (> 630 µm), 14 ± 2 (> 250 µm), 44 ± 2 (> 125 µm), 145 ± 15 (> 75 µm) and 299 ± 27 (> 25 µm). The %DIF of detected MPF per particle size varies between the two methods, depending on the particle size and range from 11% (> 125 µm) up to 480% (> 25 µm). For particles larger than 125 μm, detections are similar between NRafm and cmRs. However, for particles smaller than 125 μm, cmRs shows higher MPF detections, compared to NRafm. The non-parametric Wilcoxon signed-rank test (*p* = 0.05) indicates no significant differences between both methods for MPF for all particle sizes, except for the particle size > 630 µm.

## Discussion

### Reduced organic matter removal by Fenton's Reagent increases false positive MP observations

The results of the present study show disparate MP counts between NRafm and cmRs in 92 out of 100 cases and an overall %DIF of 421%, attributed to higher MP observations detected through NRafm. Stanton et al. (2019) described similar observations when comparing NRafm with detections obtained by another dye (4′,6-diamidino-2-phenylindole) that binds to biological materials. They found that the use of NRafm alone can lead to over-predictions in MP abundance ranging between 10.8 and 100%. De Guzman et al. (2022) further reported NR staining-induced over-predictions by determining the difference in the number of MP counts between NR staining and micro-FTIR spectroscopy. They calculated the overestimation as a percentage difference based on NR-stained MP counts and found numbers ranging from 17.9 to 686.2%, primarily attributed to undigested biological residues from Manila mussels.

**Fig. 4:**
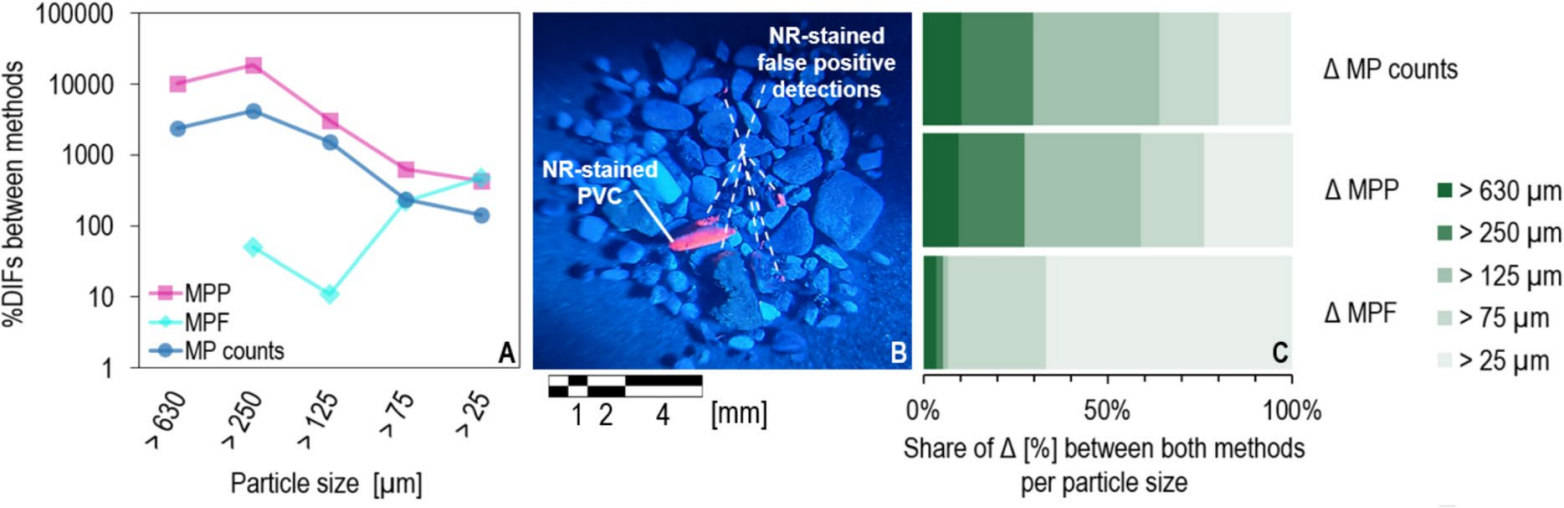
**A** Percentage differences (%DIFs) for MP, MPP and MPF counts. **B** Smartphone recorded fluorescence image of an NR-stained environmental sample showing NR-stained PVC (confirmed using cmRs) and false positives caused by residues of organic matter using a blue LED flashlight and a magnifying glass equipped with a blue bandpass filter. **C** The share of Δ (%) distributions for MP, MPP and MPF counts across five selected particle size

Beyond this, the present study reveals a particle size fraction related %DIF in the number of detected MP counts, show a linear decrease from larger to smaller particle size fractions (*R*^2^ = 0.637) (Fig. 4A). To explain this, Maw et al. (2022) demonstrated high Fenton reagent-based organic matter removal efficiency, ranging between 81.5 and 87.1%, for loamy and muddy sludge from a wastewater treatment plant, which partially covers the smaller particle sizes of the present study. In addition, consistently high organic matter removal efficiency by Fenton’s reagent applied to fine and/or suspended organic contaminants and textile dyes from wastewater has been demonstrated (Barbusiński & Filipek, 2001; Ebrahiem et al., 2017; Hurley et al., 2018; Jain et al., 2018; Liu et al., 2007; Pérez et al., 2002; Tagg et al., 2017). Although standardised protocols are lacking, we assume that the Fenton protocol applied in this study is capable of efficiently removing organic matter residues in the smaller particle size fractions, contributing to the lower observed %DIFs for smaller particle size fractions.

However, the efficiency of organic matter degradation across different particle size fractions remains poorly understood, especially for larger particle size classes. Fluorescence images recorded via smartphone (Fig. 4B) in this study clearly illustrate that Nile Red-stained organic matter residues remain in the sample after application of the Fenton reaction, indicating less efficient degradation for larger particle size fractions. This likely leads to the higher %DIF observed for the larger particle size fractions.

In summary, a size-dependent decrease in %DIF in the number of detected MP items is observed, which is likely influenced by uneven removal of organic matter by the Fenton reaction across different particle size fractions. This suggests that organic residues affect detection efficiency to varying degrees depending on particle size, which limits the result accuracy.

**Fig. 5:**
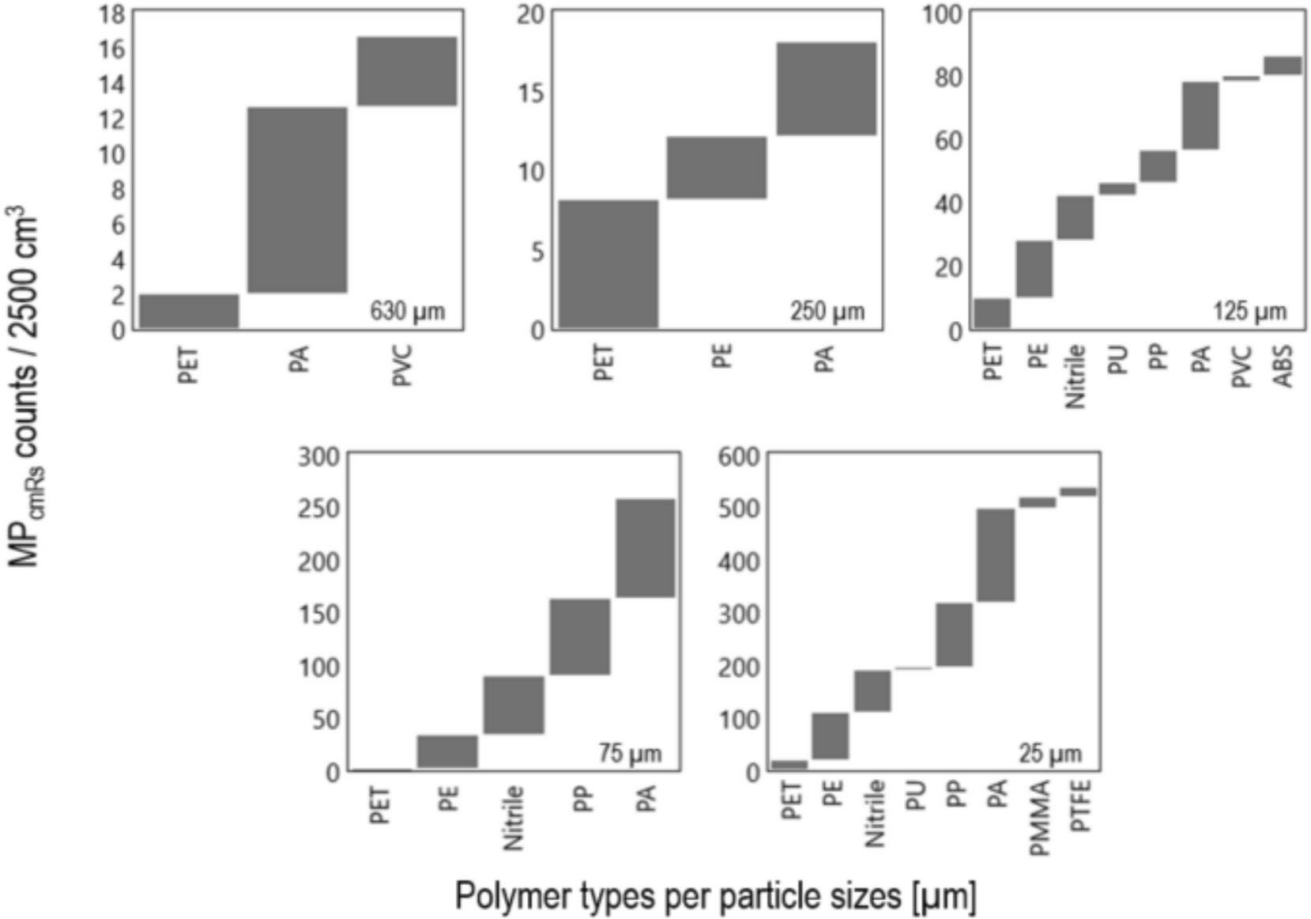
Total number (*n* = 100) of detected microplastic (MP) types for all selected particle sizes (5000–25 µm) qualified using cmRs

The polymer composition of MP fibres and particles reveals distinct patterns, with fibres being predominantly composed of polyamide (PA, 57.6%) with lower proportions of nitrile (22.0%) and polyethylene terephthalate (PET, 10.2%). In contrast, MP particles consist mainly of polypropylene (PP, 36.5%) and polyethylene (PE, 34.6%), with a broader range of additional polymer types such as PET, Nitrile, PVC, ABS, PU, PA, PMMA and PTFE. A particle size fraction-dependent distribution is evident in both MP fibres and particles. MP fibres primarily occurred in the smaller particle size fractions below 250 µm, with PA being the dominant polymer type across all fibre particle size classes. PA was particularly prevalent in the 125, 75 and 25 µm fractions, where it accounted for over 50% of the identified polymers. In contrast, MP particles showed a more heterogeneous polymer composition (Fig. 5). PE and PP were most abundant in the 125 to 25-µm particle size fractions, while polyvinyl chloride (PVC) dominated the largest particle size fraction at 630 µm. However, the analysis of the distribution of the detected polymers across the particle size fractions does not indicate that specific polymer types influence the observed %DIF between the two methods in this study.

### Morphological particle analysis reveals impact of Nile Red-assisted confocal microscopy to MP results quality

Aside from organic residuals remained in the pre-treated samples after the application of the Fenton reagent-based digestion protocol, contributing to misinterpretations and the generation of NR-stained false positive MP detections, three methodological factors (1–3, see below) were identified that cause morphological characteristic-related %DIFs in MPP and MPF counts between both methods. The detection performance of cmRs shows similar detections for MPP and MPF counts with a %DIF of 27%. The detection performance of NRafm shows different detections for MPP and MPF counts with a %DIF 2671%, which is a remarkable gap, compared to the cmRs (Fig. 2B, C). The combination of NR staining and confocal microscopy poorly detects MPFs, as further evidenced by the stagnating number of MPF observations for particle sizes smaller than 125 µm, in contrast to MPF observations obtained using cmRs. These findings indicate that the ability to detect morphological features using the applied NR staining protocol can strongly affect the quality of the results.

**Fig. 6:**
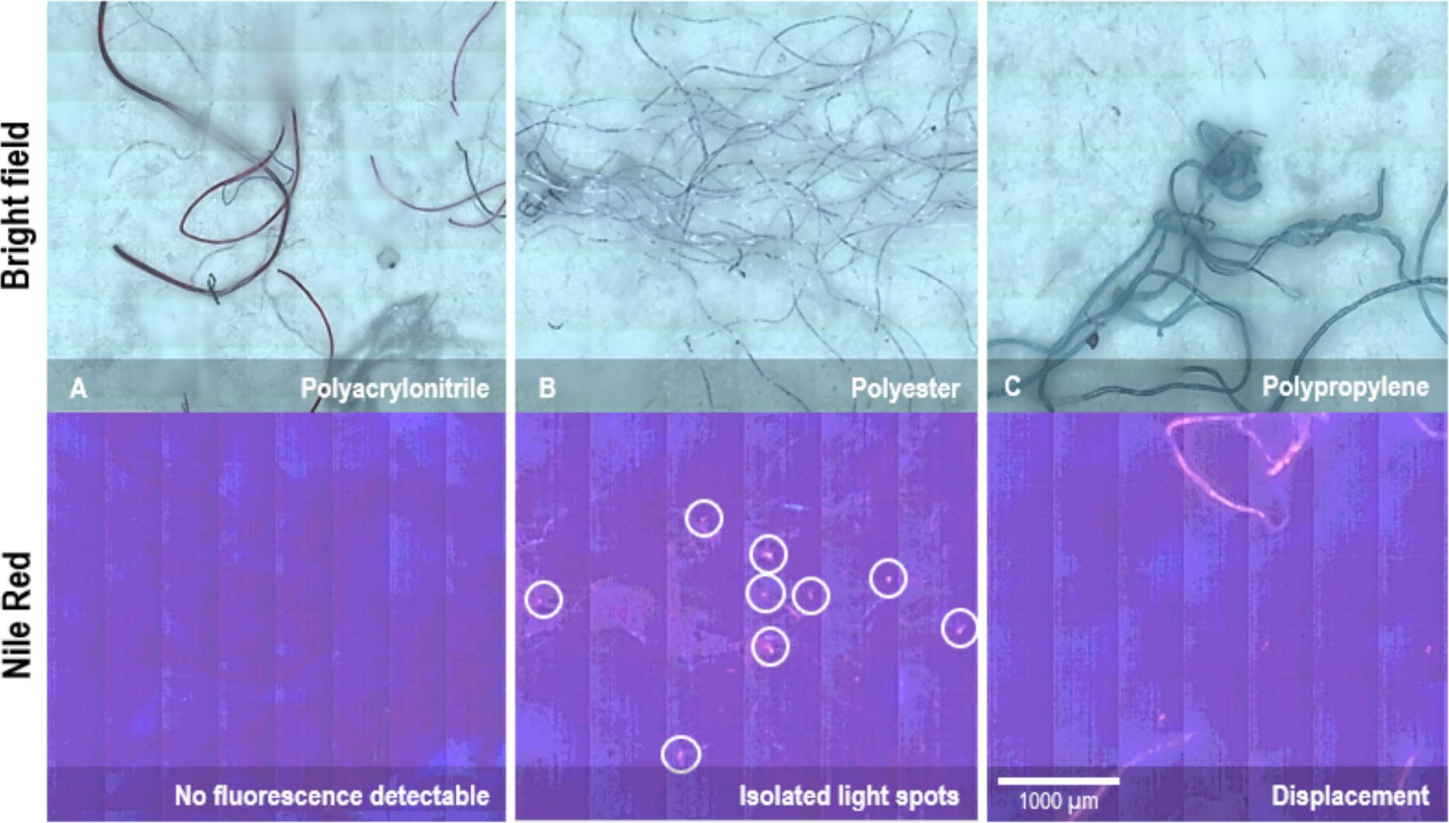
Confocal microscopic bright field images (top) and confocal fluorescence microscopic images (bottom) of the same imaging sequence prepared for demonstrating sources of misinterpretations in the results for **A** polyacryl-nitrile (PAN), **B** polyester (PET) and **C** polypropylene (PP) fibres

1. The NR-stained method was unsuccessful in detecting hydrophobic polyacrylonitrile (PAN) fibres, which are known, along with other plastic polymers such as polyethylene terephthalate (PET), polycarbonate (PC), polyurethane (PUR) and polyvinyl chloride (PVC) to emit inherently weak fluorescence signals when analysed with NRafm (Erni-Cassola et al., 2017; Karakolis et al., 2019). No fluorescence signals were visible for PAN fibres, underscoring methodological limits in the MP detection (Fig. 6A). The weak fluorescence of hydrophobic polymers, combined with the use of blue LED flashlights positioned around the confocal microscope, may not provide sufficient excitation energy to detect fluorescence signals effectively, as the light of blue LED flashlights is broadly scattered before reaching the sample. Karakolis et al. (2019) suggested that using alternative textile dyes, such as iDye Poly or Rit DyeMore Synthetic, could produce more intense fluorescence signals. When coupled with controlled heat application and longer exposure times (24 h) during staining, these protocols have been shown to yield stronger fluorescence, potentially enhancing the detection of NR-stained hydrophobic polymers using the methodological setup employed in this study.
2. Inadequate depiction of NR-stained MPF resulted in a fluorescence image where isolated light spots are detected instead of a complete NR-stained fibre (Fig. 6B). These isolated spots are likely due to the accumulation of NR at the fibre ends, causing more intense light emissions, compared to the rest of the fibre (e.g., Cole, 2016; Erni-Cassola et al., 2017; Karakolis et al., 2019). This phenomenon may lead to the misclassification of MPFs in the present study.
3. Displacements of polypropylene fibres following the dropwise application of the NR solution (Fig. 6C), potentially leads to the transportation of MPP and MPF beyond the mapping area of the fluorescence images. This displacement could contribute to inaccuracies and misinterpretations in the detection of MP in the present study.

### Smaller grain sizes lead to higher uncertainty during extrapolation

**Fig. 7:**
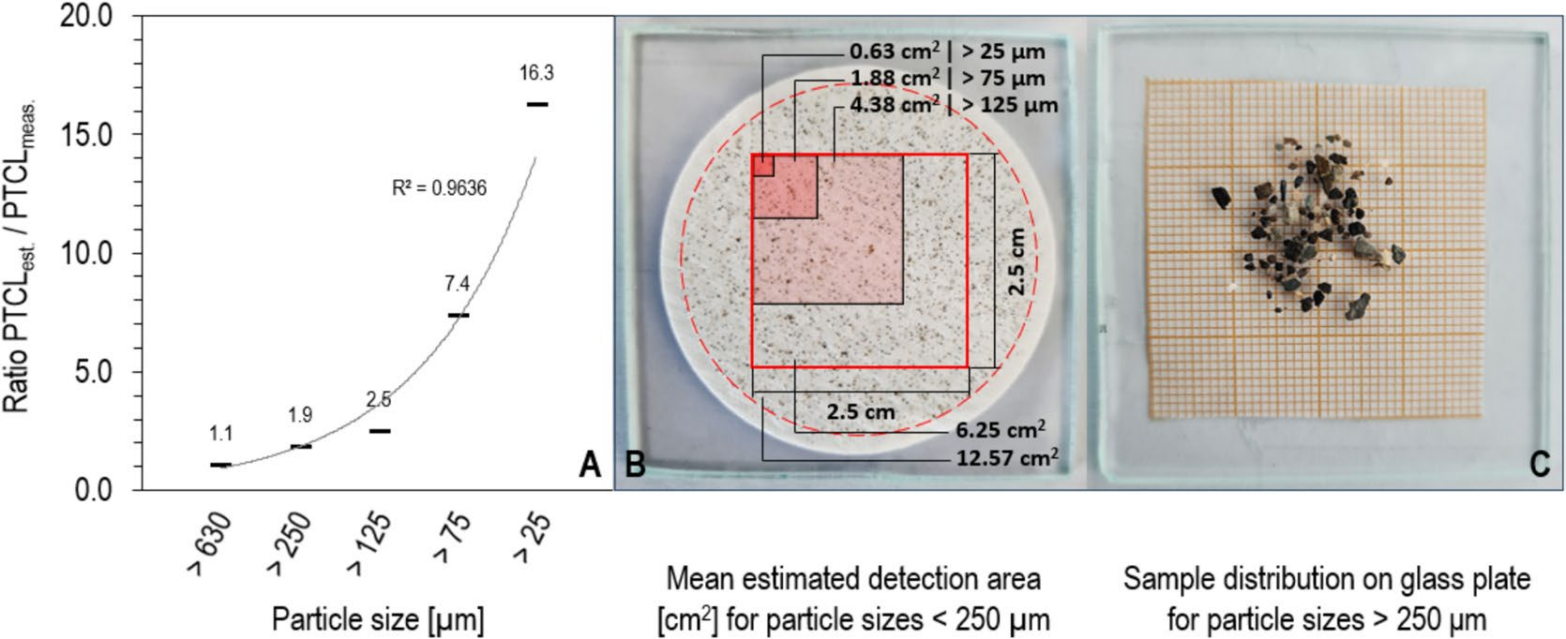
**A** Exponentially increasing (*R*^2^ = 0.9636) ratio between total estimated (est.) and measured (meas.) particles (PTCL). **B** Mean mapped detection area (red shaded) per particle size (µm) and the total microscopically recorded detecting area (red outlined) with 6.25 cm^2^. The membrane in the background (red dashed line) shows a homogenously distributed sample (> 125 µm) transferred by using vacuum filtration. **C** Distributed sample (> 250 μm) on the glass plate equipped with graph paper

Approximately 400–500 objects were measured per filter membrane within the mapping area. Reducing the number of objects for the Raman measurements to analyse multiple mapping areas per filter membrane, for example, to obtain a more robust average of the objects on a filter membrane, would have significantly increased measurement times, as all particles were manually selected. This limits the number of objects examined on the filter membrane in this study. The automatic detection of objects on the filter membranes using GEPARD (Gepard-Enabled PARticle Detection) for Raman microscopes could facilitate the analysis of more objects across multiple mapping areas, provided the sample is homogeneously distributed on the filter membranes, and the objects do not overlap with each other (Bittrich & Brandt, 2018). Additionally, linearly extrapolating cmRs-based MP detections (< 250 µm) to the entire filter membrane introduces increasing uncertainties in the MP observations, as the area of detection decreases with smaller particle sizes and no data is available for validation outside the detection area (Fig. 7B) (Käppler et al., 2016). As the particle size decreases, the ratio between the total estimated particles on the membrane and the measured particles rises exponentially (Fig. 7A), compromising the robustness of the extrapolation. The difference between the number of estimated particles and the number of measured particles spans over two orders of magnitude, particularly for particle sizes smaller than 125 µm, underscoring the impact of the increasing ratio on these fractions. Enhanced comparability of the extrapolated results can be achieved by aligning the Nile Red mapping areas with the average Raman mapping areas (Fig. 7B). Additionally, the extended measurement times required for cmRs limits the processing of larger sample quantities. In this study, only two membranes could be analysed between 6 a.m. and 4 p.m. In contrast, the number of NR-treated membranes analysed within the same timeframe was substantially higher, emphasising the importance of co-applying complementary MP detection techniques.

Standardised laboratory tests can help identifying limiting factors related to MP grain size and morphology that contribute to the observed percentage differences between cmRs and NRafm. The discussions of the percentage differences between both methods could form the basis for generating potential boundary conditions in future investigations under controlled laboratory conditions, focussing on possible sources of analytical error in a dual-method MP detection approach for high-throughput analysis. This, in turn, holds the potential to improve the precision, accuracy and reliability of MP detection in environmental samples by minimising methodological biases and enhancing reproducibility.

## Conclusions

The study highlights that differences in MP detections between NRafm and cmRs occur in 92% of the cases. While the present dual-method MP detection approach significantly enhances sample throughput, it also highlights the need for further refinement to mitigate uncertainties and potential misinterpretations in quantitative and qualitative MP analyses. Key findings are:

- Particle size influences the detected percentage differences and reveals that both MP detection methods deliver better matching results at smaller particle sizes.
- Detected plastic types show no significant influence on the observed percentage differences between the two methods.
- Challenges in degrading organic matter and reproducing the morphological characteristics of the detected MP between both methods highlight the urgent need for better organic matter removal techniques for larger objects (> 75 µm) and a better understanding of organic solvent-induced degradation of smaller MPs (< 125 µm).
- Overcoming the susceptibility of Nile Red staining to the detection of false positives (incompletely removed natural organics) by combining the Nile Red staining approach with Raman spectroscopy’s reliability to distinguish organic material from MP, highlights the potential for cross-validation and data correction between of both techniques.

Future studies should prioritize the application of multiple MP detection techniques to enhance sample throughput and data validity. The development of effective protocols for removing larger-sized organic matter from samples and understanding MP degradation caused by the exposure to organic solvents during sample preparation is a necessary step for an improved understanding of MP fate in the environment. Data validation to enhance precision and reliability is essential, particularly when applying Nile Red staining for MP detection in natural systems.

## Data Availability

Data availability The data used in this study are available upon request from the corresponding author.
